# A Comprehensive Open-Source Simulation Framework for LiFi Communication

**DOI:** 10.3390/s21072485

**Published:** 2021-04-02

**Authors:** Shakir Ullah, Saeed Ur Rehman, Peter Han Joo Chong

**Affiliations:** 1 Department of Electrical and Electronics Engineering, Auckland University of Technology (AUT), Auckland 1010, New Zealand; peter.chong@aut.ac.nz; 2 School of Science and Engineering, Flinders University, Adelaide 5000, Australia; saeed.rehman@flinders.edu.au

**Keywords:** VLC simulation, LiFi, VLC, *ns-3*, 6G, WiFi, simulation tool

## Abstract

Light Fidelity (LiFi) is a new candidate for wireless networking that utilizes the visible light spectrum and exploits the existing lighting infrastructure in the form of light-emitting diodes (LEDs). It provides point-to-point and point-to-multipoint communication on a bidirectional channel at very high data rates. However, the LiFi has small coverage, and its optical gain is closely related to the receiver’s directionality vis-à-vis the transmitter, therefore it can experience frequent service outages. To provide reliable coverage, the LiFi is integrated with other networking technologies such as wireless fidelity (WiFi) thus forming a hybrid system. The hybrid LiFi/WiFi system faces many challenges including but not limited to seamless integration with the WiFi, support for mobility, handover management, resource sharing, and load balancing. The existing literature has addressed one or the other aspect of the issues facing LiFi systems. There are limited free source tools available to holistically address these challenges in a scalable manner. To this end, we have developed an open-source simulation framework based on the network simulator 3 (*ns-3*), which realizes critical aspects of the LiFi wireless network. Our developed *ns-3* LiFi framework provides a fully functional AP equipped with the physical layer and medium access control (MAC), a mobility model for the user device, and integration between LiFi and WiFi with a handover facility. Simulation results are produced to demonstrate the mobility and handover capabilities, and the performance gains from the LiFi-WiFi hybrid system in terms of packet delay, throughput, packet drop ratio (PDR), and fairness between users. The source code of the framework is made available for the use of the research community.

## 1. Introduction

Over the last two decades, there has been exponential growth in the number of mobile devices. According to the CISCO visual network index (CVNI), by 2023, around 70% of the world population will have mobile phones [1]. The increase in mobile phones is coupled with an emphasis on exploring new use cases for mobile/cellular networks like the internet of things (IoT) to enable smart homes and smart cities, including vehicular communication [1]. This rapid expansion in size and scope of mobile networks is expected to increase data consumption exponentially. The CVNI reports that around 48% of IoT traffic and around 80% of other mobile traffic will originate from the indoors [2]. Similar trends in data consumption have been predicted for outdoor mobile networks. To support a large number of devices and to meet the growing traffic demands, the wireless research community has been looking for additional spectrum in the higher frequencies band, such as the millimeter waves (for 5G) in the range of 30 to 300 GHz. However, the increased use of the radio spectrum can result in increased interference, which can drive down the throughput. On the other hand, visible light communication (VLC), which relies on the visible light spectrum in the 430 THz to 730 THz range, is a promising candidate in the quest for additional spectrum in the high-frequency range. The VLC’s higher frequency spectrum ensures high data rates, even over 1 Gbps [3]. Besides, it can provide secure communication as the visible light cannot penetrate walls and can thus be confined to a room to avoid eavesdropping. The wide deployment of lighting infrastructure in the form of LEDs further motivates the use of VLC. More importantly, the LiFi/VLC can co-exist in an interference-free manner with WiFi and cellular technologies such as LTE and 5G. These benefits put LiFi as a prime candidate for additional wireless communication technology in indoors and outdoors. In the indoors, LiFi is expected to be integrated with WiFi to increase its capacity, provide low latency and higher throughput [4,5,6]. On the other hand, in the outdoors, the LiFi communication can be integrated with 5G to enable smart cities, particularly vehicular communication where low-latency safety-critical information can be delivered through the visible light medium [7,8,9].

**Figure 1:**
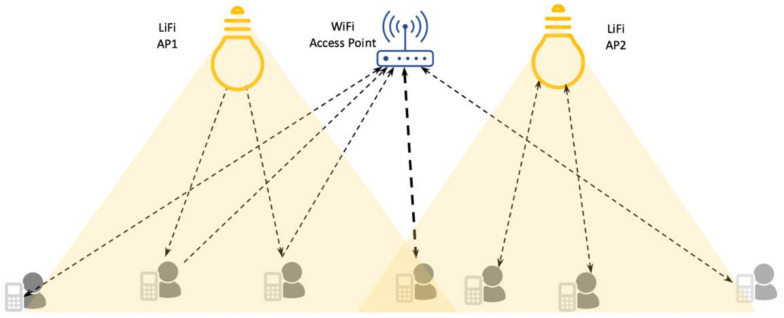
Hybrid light fidelity/wireless fidelity (LiFi/WiFi) system.

The advantages of VLC have been harnessed in the form of LiFi, which provides a complete wireless networking system [3]. The LiFi promises to use LED lights as access points (APs) and provide bidirectional high data rate communication and multiuser access with dynamic user associations. It is also expected to offer seamless mobility and interoperability with other networking systems such as WiFi and long-term evaluation (LTE) or 5G [3]. However, the LiFi has a higher penetration loss, and a significant portion of its gain comes from the line of sight (LOS) communication, making it difficult to be used as a standalone system. Therefore, the LiFi is a complementary technology to WiFi, thus forming a hybrid networking system. The hybrid system of LiFi and WiFi can be realized in two ways: Symmetric and asymmetric, as shown in Figure 1. In asymmetric hybrid systems, the downlink of LiFi is provided through VLC and the uplink is supported via WiFi. In symmetric hybrid system, the LiFi provides bidirectional communication in which the VLC provides the downlink communication, and the infrared provides the uplink. The hybrid network of LiFi/WiFi offers consistency in coverage, increase in capacity, and lower latency. 

Although LiFi offers many advantages including the readily available infrastructure in the form of LED lights, it still needs significant efforts to address issues such as optimization of physical and MAC layer resources, handling user mobility, including the provision of seamless handover. The existing work has focused on many of these challenges; for example, in [10], the LiFi resources are shared among multiple users using the optical orthogonal frequency division multiplexing (OFDM). Miramirkhani et al. have modeled power and delay profiles of mobile VLC and provided a power adjustment mechanism to optimize gain [11]. In [12], the authors carried out experimental work for mitigating spatial and temporal crosstalk in optical MIMO systems by using decoding algorithms and equalizers, respectively. In [13], the authors present the theoretical concepts and techniques involved in developing MATLAB models for a short-range 4 × 4 multiple-in multiple-out (MIMO) VLC system. Liverman et al. [14] have developed a system called WiFo, integrating free-space optical communication (FSO) and WiFi system to provide higher throughput with seamless handover. Shen et al. [15] have explored the effects of road surface irregularities on SNR performance of the VLC in the context of vehicular communication. Guzmán et al. [16] have analyzed the OFDM performance for outdoor VLC in the presence of sunlight. The authors in [17] have created a hybrid WiFi and LiFi system to provide LiFi and WiFi aggregated communication channels. The authors in [9] have developed a VLC enabled system for low-latency, safety-critical intelligent transportation systems (ITS). Their system can deliver information under sub-millisecond up to 30 m, and under 10 ms for a distance of 50 m with 99% confidence. In [8], experimental work has been carried out for joint 5G and VLC vehicular information delivery system. It is reported that 5G can achieve consistent latency of 9.5 ms and 2.5 ms for VLC. Khreishah et al. [18] have proposed a framework for energy-efficient AP selection in VLC and WiFi hybrid systems. The LiFi channel is modeled with different modulation and coding rates are discussed in [19]. In [20], the user mobility and device orientation change effects on key performance metrics are explored. In [4], integrated orthogonal frequency division multiple access (OFDMA)-based LiFi and WiFi systems are explored for load balancing and handover for mobile users. To avoid excessive handovers in an AP, a selection method is proposed by serving users simultaneously from multiple APs [21]. The authors in [5] have proposed a joint user-centric coordination multipoint (CoMP) to improve space diversity gain and thereby improve the signal-to-noise-to-interference ratio (SINR).

The literature on LiFi is mostly related to optimizing the physical and MAC resources and mobility and network association schemes [4,8,9,14,15,16,17,18,19,20]. Most of the existing work utilizes bespoke simulation platforms to evaluate and analyze their protocols and research work [3]. Moreover, these studies have focused on evaluating their solutions in limited environments containing few access points, thereby lacking the scalability aspect. On the other hand, it is expected that the addition of LiFi to the hybrid systems will increase the number of APs making it necessary to evaluate any new algorithm or protocol on simulation platforms that can provide scalability. In this regard, many modern-day simulators, such as *ns-2 ns-3*, OMNET++, and GloMoSim are equipped with tools that allow for the simulation of large-scale networks that can be easily reproduced in the real-world environment. The capabilities offered by these tools are not fully exploited to provide a comprehensive simulation platform for LiFi. Among the available networking simulators, we choose *ns-3* for developing our framework for the following reasons:

- The *ns-3* has a vibrant research community in terms of researchers, developers, and users [22].
- It is memory efficient compared to other networking simulators such as OMNET ++, GloMoSim and *ns-2* [23].
- Protocols from *ns-3* could be easily reflected in and integrated with real networks [24].
- It has native modules for LTE and WiFi, making it easy to develop a hybrid system with LiFi [25]. There are many third part 5G new radio (NR) modules that could be integrated with LiFi to help simulate heterogeneous 5G networks [26].

To harness the above advantages, this work provides a comprehensive implementation in *ns-3* for the LiFi networking technology, including its integration with WiFi. To the best of the author’s knowledge, the closest simulation tool in this regard is reported in [27], which consists of the VLC module for *ns-3*. The proposed work provides point-to-point communication, error, and SNR models. However, it cannot provide fully functional LiFi APs capable of providing multiuser access, offer bidirectional communication, support for user mobility with orientation changes, handover facility, and integration with WiFi. Our developed open-source framework implements all the critical features of a scalable LiFi-WiFi system. It provides a physical layer, a MAC layer implementation in the form of TDMA, the ability to associate users with the AP, mobility modeling, a handover scheme between LiFi and WiFi APs, and a centralized controller to store information about the networking devices. To validate our simulation framework, we have evaluated the LiFi system’s performance in terms of throughput, delay, and fair sharing of mobile users. The proposed simulation framework has been made available as an open-source code to increase the LiFi researchers’ productivity [28]. 

The rest of the paper is organized as follows: Section 2 starts with a conceptual overview of the framework. Section 3 provides a brief discussion about the mathematical foundations of the LiFi system. The *ns-3* design of the LiFi framework is presented in Section 4. The integration between LiFi and WiFi systems including the handover process is discussed in Section 5 and Section 6, respectively. The evaluation metrics are discussed in Section 7.

Section 8 describes the simulation setup and the results. In Section 9, we conclude our work. 

## 2. Conceptual Overview of the LiFi Simulation Framework in *ns-3*

In this work, we have implemented an *ns-3* framework for LiFi and provided its integration with the WiFi. First, we provide necessary mathematical descriptions of the critical aspects of the LiFi physical and MAC layers. For the physical layer, we describe the bidirectional LiFi channel, mobility, and packet reception metrics (e.g., SNR, BER). At the MAC layer, we provide multiuser access using time division multiple access (TDMA) with resource sharing capabilities and user association/reassociation with the APs. The mathematical description of LiFi is then developed into *ns-3* designs with accompanied algorithms and their implementation. Once the physical and MAC layers LiFi are realized, we discuss its integration with the built-in *ns-3* WiFi module based on the IEEE802.11 standard. The integration includes, among other techniques, a handover scheme that monitors critical performance metrics for LiFi users for a certain threshold and switches the networks accordingly.

## 3. LiFi System Model

The LiFi systems at the most fundamental level consist of LED transmitters that employ the principle of intensity modulation to convert the incoming electrical signal to information before transmission through the optical channel. The LED transmitter can register new users and serve multiple users over the same optical channel thus forming a LiFi access point (AP). The receivers are equipped with photodetectors (PD), which convert the falling light to optical current. Many of the modern receivers are mobile and equipped with the necessary hardware to model the mobility aspect of the LiFi system and analyze its effect on receiver gain. From the receiver gain and the channel, an accurate error assessment can be made for packet reception. A detailed mathematical model of the underlying components of the LiFi systems is discussed below. In Section 3, the mathematical models are implemented into an open-source *ns-3* framework. 

### 3.1. Physical Layer

The LiFi physical layer represents the over-the-air interface including the channel, modulation and coding schemes, and packet reception metrics. For the channel, we consider the optical channel consisting of the LOS and non-LOS (NLOS) components. For the modulation, we consider the basic modulation schemes such as pulse amplitude modulation (PAM) and on-off keying (OOK) and advanced modulations such as quadrature amplitude modulation (QAM). The coding schemes are left as future work. The packet reception at the physical layer is modeled using the signal-to-noise ratio (SNR) and bit error rate (BER) models that are based on the considered modulation schemes. Below we provide the details of the physical layer aspects of the LiFi systems.

#### 3.1.1. Channel Model

The LiFi channel consists of a LOS component that communicates through a direct path over the air, and the non-LOS (NLOS) paths that can be formed due to the reflections from the walls, ceiling, and other objects. The LiFi system predominately relies on the LOS gain; however, it has been demonstrated in [29] that at certain positions in the room, the gain from the NLOS channels can be enough to provide communication. 

The LiFi channel gain is affected by multiple factors such as the angle of arrival (AOA), which is heavily affected by the user device orientation, the angle of emission at the transmitter ($T_{x}$), and the user device field of view (FOV). For multiple LiFi APs supporting multiple users, the gain for a user *k* from a particular AP *i* along the LOS component can be calculated as below [29].

(1) $$H_{LOS}^{i,k}=\;\frac{\left(m_{l}+1\right)A}{2\pi {\left(h+d\right)}^{2}}\;{cos}^{m}\;\left(\emptyset \right)\;T_{x}\;g\;\left(\psi_{i,k}\right)cos\left(\psi_{i,k}\right)\;\Pi \left(\psi_{i,k}/\psi_{con}\;\right)$$

where $H_{LOS}^{i,k}$ is the LOS of user *k* from AP *i*, h is the vertical distance of the user device from the AP, d is the horizontal distance on the floor, m is the Lambertian order of emission, A is the PD area, and g $\left(\psi_{i,k}\right)\;\mathrm{is}\;\mathrm{the}\;\mathrm{optical}\;\mathrm{concentrator}\;\mathrm{gain}.$ The Lambertian order is given as $m\;=\frac{-ln\left(2\right)}{ln\left(2\right)\left(\cos\left(\Phi_{1/2}\right)\right)}$, where $\Phi_{1/2}$ is the semi-angle at half transmit power. The function $\Pi \left(\psi_{i,k}/\psi_{con}\right)$ represents whether device orientation is within the field of view (FOV) of AP. For within FOV the $\Pi \left(\psi_{i,k}/\psi_{con}\right)$ < 1 it is 1, and 0 otherwise. Equation (1) above ∅ represents the angle of irradiance of the transmitter, and ψ represents the AOA of the receiver. Both these angles can be calculated for access points/transmitters installed at position ($x_{t},\;y_{t},z_{t}$) and the receiver located at position ($x_{r},\;y_{r},z_{r}$) as below [20,30,31]:

(2) $$\cos\left(\emptyset \right)=\frac{-n_{tx}*d}{d}$$

where *n_tx_* is normal vectors to the transmitter, *d* is the distance vector between the transmitter and user device, and ***d*** is the Euclidean distance between the transmitter and the receiver. The cosine of AOA can be calculated as below [20]:

(3) $$\cos\left(\psi \right)\;=\;\frac{\left(x_{t}-x_{r}\right)}{d}\sin\left(\theta \right)\cos\left(\omega \right)\;+\frac{\left(y_{t}-y_{r}\right)}{d}\sin\left(\theta \right)\cos\left(\omega \right)\;+\frac{\left(z_{t}-z_{r}\right)}{d}\cos\left(\omega \right)$$

where ω is the angle between the projection of the user device normal *n_u_* along the positive axis on xy-plane, and θ is the angle between *n_u_* and the *z*-axis. More discussion about how to produce angle θ is provided in Section 3.2.

For the NLOS channels, we consider the first-order reflections from the walls, which are assumed to arrive simultaneously at the receiver [32]. The reflected arrays have two paths: First, from AP to the small area on the wall represented as wall area (WA); second, a reflected path from WA to the user device [32].

(4) $$H_{NLOS}^{i,k}\;=\overset{ℜ}{\underset{j=1}{\sum }}\frac{\left(m_{l}+1\right)\rho_{j}\Delta WA}{2\pi \;d_{S,j}^{2}d_{R,j}^{2}\;}\;cos^{m_{l}}\left(\emptyset_{Sj}\right)cos\left(\Psi_{Sj}\right)cos\left(\Psi_{Rj}\right)$$

where $d_{S,j}^{2}$ is the distance from the source AP to the WA, $d_{R,j}^{2}$ is the distance of point source on the wall to the user device, ℜ represents the number of reflection sources, and $\emptyset_{Sj}$ is the angle of transmission between the source and reflection j. In Equation (2), $\Psi_{Sj}$ and $\Psi_{Rj}$ indicate the angle of incidence at the point receiver on the wall and the user device, respectively. The values for these parameters are specified based on [29,33]. The total gain from the LiFi channel can be calculated as below.

(5) $$H_{i,k}=\;H_{LOS}^{i,k}+H_{NLOS}^{i,k}$$

The uplink and downlink channels’ symmetry allows us to use (1–4) for both the downlink and uplink. 

Our open-source simulation framework has implemented only the first-order reflections from the walls and ceiling. The research community can add higher-order reflections to the existing framework trivially.

#### 3.1.2. Packet Reception Metrics

At the LiFi physical layer, we evaluate packet reception based on the signal-to-noise ratio (SNR) and the error models. The packet reception depends on whether SNR is above a certain threshold, which depends on the optical gain of the channel, the PD responsivity, and the noise.

(6) $$SNR=\frac{P_{r\;}^{2}r^{2}}{\sigma^{2}}$$

where $P_{r\;}^{2}$ is the received optical power, which can be obtained by dividing the channel gain given in Equation (5), over the squared distance of the transmitter and the receiver, $r^{2}$ is the responsivity of the PD, and $\sigma^{2}$ is the total noise. For noise, we consider the thermal noise coming from the electronics in the user device and transmitter, and the ambient noise from the natural lighting.

The error probability or bit error rate (BER) performance for LiFi networks can be evaluated in terms of SNR and the modulation schemes. For some of the commonly used modulation schemes, the bit error performance is listed below. For on-off keying (OOK) and pulse amplitude modulation (PAM) schemes, the BER can be calculated as below [27].

(7) $$BER_{PAM}=\frac{2*\left(M-1\right)}{M}Q\left(\sqrt{\frac{SNR}{M-1}}\right)$$

where *Q* is the tail probability of the normal function, and M is the modulation order. As the OOK is a subset of PAM its order is kept as 2. The packet corruption can be determined from the BER value as below [27].

(8) $$PER_{PAM}=1-{\left(1-BER_{PAM}\right)}^{\frac{8*p}{\log2\left(M\right)}}$$

### 3.2. Mobility Modeling

The LiFi is expected to support mobile devices with strict QoS requirements in terms of delay, reliability, and throughput. Since the LiFi channel gain fluctuates significantly with changes in user movement or with device orientation changes. These changes in channel gain can be significant enough to cause service outages and can, therefore, negatively affect critical QoS metrics. Therefore, it is of paramount importance to provide accurate mobility models that can be used as a basis to associate users with an accurate network and perform handovers in case of the service outage. Fortunately, in this regard, most modern mobile devices are equipped with the necessary hardware in the form of a gyroscope and accelerometer to assess user mobility. 

In our simulation implementation, user mobility encapsulates both the user movement and device orientation changes. The user movement pattern is assumed to be random in an area bounded by dimensions $D_{max}\;\mathrm{and}\;D_{min}$ with a step size of $D_{k}$, uniformly distributed over the bounded area $\mu \left(D_{k}\right)=\left[D_{max},D_{min}\right]$. The random movement affects ω angle in the positive direction along xy-plane, and the vertical angle θ of a mobile device with the AP, normally distributed with a mean $\theta_{\mathrm{mean}}$. We adopt the *ns-3* random waypoint mobility model for modelling user mobility, which provides support for random user (bounded or unbounded) movement. To add the random user device orientation aspects, we adopt the work in [20], which provides a statistical model based on real subjects’ movement in a typical indoor scenario. A mobile device’s random orientation can be represented as a correlated Gaussian random process with a mean of 29.67 and variance of 7.78. The correlated samples for the user device orientational θ can be generated using the first-order autoregression model (AR) as below [20]:

(9) $$\theta \left[n\right]\;=c0+c1\theta \left[n-1\right]+w\left[n\right]$$

where $w\left[n\right]$ is a white noise process with the variance of $\sigma_{w}$. The coefficients c0, c1, and the variance $\sigma_{w}$ for the random process are generated as below [20].

(10) $$c0=\;\left(1\;-\;c1\right)E\left[\theta \right],\;c1=0.005^{\frac{Ts}{Tc,\theta ,}}E\left[\theta \right],\;\sigma_{w}=\;\left(1-c1^{2}\right)\sigma_{\theta }$$

where T_s_ is the sampling time and $T_{c,\theta }$ is the coherence time of the random process θ. These representations along with algorithms presented in [20] are adopted for the random waypoint mobility model in *ns-3.*

### 3.3. AP Design with Multi-User Access Using TDMA

The LED-based light bulbs can be offered as APs by equipping them with capabilities such as multiple user access and dynamic user association/reassociation. The multiple-user access can be provided by sharing the AP resources (e.g., optical channel and queues) between users via MAC protocols such as time division multiple access (TDMA), carrier sense multiple access with collision avoidance (CSMA/CA), and code division multiple access (CDMA). On the other hand, dynamic user association is achieved with the modification to the MAC layer through provisions of protocols such as beacon framing or association request/response mechanism. 

Our simulation framework provides an implementation of multiuser access through TDMA that shares the same physical medium in time. Generally, in TDMA, the same frequency channel is divided into multiple time slots: The friction of time allocated to a user to transmit or receive data in the uplink or downlink. The slot’s time is determined by the amount of channel bandwidth and the deployed modulation scheme. Each user can have one or more of these slots and is allowed to transmit or receive data only in those time slots. 

The TDMA slots are bundled into frames consisting of control slots for transmission control signal and data slots for data transmission. The control slots are used for management purposes such as advertisement of time synchronization information between the AP and the mobile stations, and beacons slots that are used for user association/reassociation. At the start of each frame, some numbers of slots are reserved to broadcast the time information of the slots allocated to each user, and at the end of each frame, the access point broadcasts beacons that contain its address and transmit power. The beacon messages are responded to by the user devices with addresses of their own and the detected SNR level. Lastly, the user device can transmit or receive actual information during the data slots. 

The LiFi APs have a small coverage area and thus they can support only a small number of users; however, they can be served at much higher data rates due to larger available bandwidth. However, higher data rate is not the only metric that needs improvement, as many of the modern applications expect diverse quality of service (QoS) requirements in terms of delay, throughput, and reliability. Besides, internet-enabled devices can have several applications running simultaneously. A device can have a Skype and Zoom video or audio call, while simultaneously having active browsing sessions and a download or upload going in the background. Video and audio calls have stringent delay and throughput requirements. On the other hand, for best-effort (BE) traffic (e.g., web browsing) and background traffic (e.g., email and file uploads), violations in throughput and delay requirements are tolerable. An AP typically must support all these services simultaneously with available time and frequency resources. We provide a heuristic scheduling scheme to distribute frame slots among users according to the aforementioned QoS requirements to support these service requirements. The QoS categories are adopted from the categories mentioned in the IEEE 802.11 standards [34]. The video and voice streams are allocated more slots compared to the background, and the best effort traffics. In a single TDMA frame, the data slots are distributed between the users according to the following piecewise equation.

(11) $$f=\left\{\begin{array}{ll} n_{vo}*t, & AC\_VO\\ n_{vi}*t, & AC\_VI\\ \;\;\;\;\;\;\;\;\;t, & AC\_BE,\;\;\;AC\_BK \end{array}\right.$$

where $n_{vo}$ is the number of slots chosen for the traffic with video streaming category, $n_{vi}$ is the number of slots for VoIP, *t* represents time slot, *AC_VI* is for access category (AC) video streaming, *AC_VO* for voice streaming services, *AC_BE* represents best-effort traffic, and *AC_BK* is for the background traffic. The total number of data slots in a single frame can be calculated based on the number of users and their traffic classes as below.

(12) $$T_{D}=vo*n_{vo}+\;vi*n_{vi}+bk+be$$

where $T_{D}$ is the total number of time slots for the data frame, vo is the number of slots per video user and $n_{vo}$ is the number of video users, vi is the number of slots per audio users while $n_{vi}$ is the total number of audio users, and bk and be represents slots for background and best-effort traffics. Apart from the data frames, the AP periodically controls frames containing all the critical information about time synchronization, and the association and feedbacks. The time allocated for the control frame is fixed, which can be represented as $T_{c}$. The total transmission time for a single frame consists of the data frame duration calculated in Equation (12), and the control frame duration and guard time. The frame transmission, $T_{f}$, can thus be calculated according to the following equation.

(13) $$T_{f}=G_{time}+(T_{D}+T_{c})*t$$

where $G_{time}$ is the guard time and $T_{c}$ is the control frame time. At the MAC layer, multiple data and controller frames and a beacon frame are encapsulated into one super-frame. The super-frame starts with the broadcast of a control frame containing information about the time synchronization (e.g., when each user can transmit or receive data), followed by data frames. At the end of each super-frame, a beacon frame is broadcasted through the downlink carrying key information about the AP. The total number of slots in a super-frame can be calculated below:

(14) $$T_{s}=T_{f}*n+I$$

where $T_{s}$ is transmission time of superframe, *I* is the interframe gap, and *n* is the total number of frames in the super-frame.

In this framework, we have only incorporated TDMA; however, other MAC protocols such as CSMA/CA, CMDA, and orthogonal frequency division multiplexing can be easily integrated. For CSMA, the *ns-3* already has a module available that can be updated with minor modifications to work with this framework. Similarly, the *ns-3* spectrum module provides frequency-dependent communication and can be modified to work as an OFDMA MAC layer protocol.

## 4. LiFi Design in *ns-3*

*ns-3* is a reliable and reputable simulation platform to develop networking protocols that can be easily realized in production networks. It is open-source with added functionalities and has a wider acceptance in the research community. The *ns-3* has a rich set of libraries and modules that can be exploited to develop and analyze new networking protocols and build large-scale networks. The core components of *ns-3* include nodes, channels, net devices, and applications. Nodes in *ns-3* are equivalent to the networking terminals such as APs, mobile phones, laptops, and servers. It can host mobility models, IP protocol stacks, and interface cards. The channel model provides generic channel functionalities but can be customized for other technologies such as WiFi and LiFi. We have customized the channel model to provide bidirectional communication. The *ns-3* net devices represent the data link layer that provides access to the channel and interfaces with the network and transport layers. It can be customized to provide multiple users access through medium access control (MAC) protocols such as TDMA, CSMA/CA, and OFDMA. The application module represents a basic abstraction that generates some activities to be simulated. In addition to these basic components, the *ns-3* provides helper classes to facilitate the installation of nodes, net devices, channels, and IP interfaces. The basic *ns-3* abstractions are composed (interconnected) to realize a network instantiation in *ns-3*. The classes for every *ns-3* module can be organized in these subfolders: helpers, models, examples, and documentation. The classes related to nodes, net devices including the MAC layer, and the physical layer are included in the model subfolder. The other subfolder consists of the classes related to the functionalities as their names indicate. For example, the helper subfolder stores files, and the example subdirectory contains use cases.

**Figure 2:**
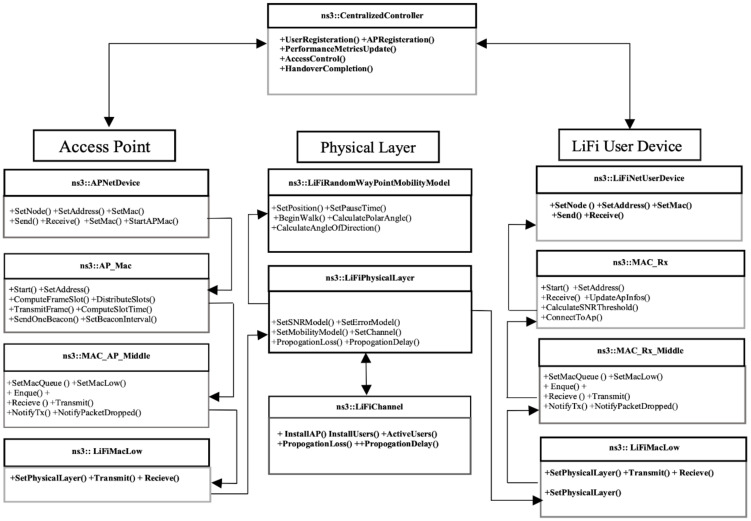
LiFi *ns-3* design.

In this work, our *ns-3* framework is based on the discussion in the previous sections. The existing *ns-3* functionalities are customized and new features are added to realize the framework. Classes from the framework are placed in the appropriate directories. The core functionalities of the critical classes are described below, and interaction between the core components of the LiFi framework are shown in Figure 2. However, a detailed description can be found in the documentation folder of the simulation framework.

### 4.1. Physical Layer

The physical layer class works in coordination with the channel model class, the SNR models, the error models, and propagation loss models for packet reception. The propagation loss class works closely with the mobility model to calculate the channel gain from Tx power, user device’s FoV, optical concentrator gain, and filter gain. The total received power is calculated from the channel gain and the noise from the thermal and ambient light sources. The SNR and the corresponding bit error are computed based on [27]. Once the packet is received in the destination device, the error model is applied to determine if it can be passed to the upper layer successfully or dropped otherwise. Lastly, we also provide traces for a packet received and changes in SNR due to user mobility. 

The LiFi channel class implements the actual transmission on the physical medium. It maintains a list of pointers to the physical layer objects of the AP and the user devices. The channel has a send method, which is activated by the sending device’s physical layer object. 

### 4.2. LiFi Mobility

In LiFi networks, the user movement and device orientation changes can significantly fluctuate the receiver gain. Therefore, to accurately accommodate for the optical fluctuations, we have adopted three different mobility models: (1) Random waypoint mobility model (RWP) with orientation changes; (2) constant velocity mobility modeling; and (3) constant position mobility model. These three models cover most of the mobility either due to user or device [20]. Nevertheless, users can implement their unique mobility patterns. 

In this work, we particularly focus on the implementation of the random waypoint mobility model, which allows a user to move around with random receiver orientations. For each movement, we calculate the angle along the direction of movement and vertical angle between the user device and AP based on the discussion in Section 2. The current position, angle of the user’s direction of movement, and the vertical angle are stored as a single measurement of mobility. During the propagation loss calculation, these measurements are used to calculate the receiver gain. In the constant velocity mobility model, the user movement along a straight line with constant speed is modeled. The current position, as well as the polar angle, is returned as a single sample of data. Lastly, the constant position mobility model can be trivially supported using the existing *ns-3* constant position mobility model.

### 4.3. LiFi MAC Layer

Based on the discussion in Section 3.3, we have implemented the TDMA MAC layer functionalities. Overall implementation of the MAC layer is divided into three layers along with the analogy of the WiFi *ns-3* module: A higher layer, a middle layer, and a lower layer. These classes are further customized to provide the AP and the user device-specific behaviors. The primary function of the higher layer classes is to provide resource distribution and user association. In the LiFi AP, the higher layer class is customized in the access point in the form of AP_Mac that provides a resource sharing algorithm. The resource-sharing algorithm schedule users on the downlink and uplink slots and send control frames. Here we provide a heuristic resource sharing algorithm that shares a common pool of data slots between the uplink and the downlink users according to their QoS requirements. As previously said, the resource sharing algorithm reserves slots for control purposes including beacon generation for user registration. To register users, the AP_Mac sends beacon (control) frames at the end of each super-frame consisting of AP’s IP address, T_x_ power, and its MAC address. The higher layer MAC class, named as Mac_Rx, in the LiFi user device is provided with a receive method that parses beacon frames, and if the SNR is above a certain threshold, it sends a response containing its MAC address and the QoS requirements. Upon reception of the user response, the AP_Mac adds them to the connected users. A scheduling algorithm then uses this information to allocate slots. A complete description of the resource sharing algorithm is provided in Algorithm 1. The middle layer queues and dequeues packets before forwarding them up or down the protocol stack. In the LiFi AP, the customized middle layer class is referred to as Mac_AP_Middle, and in the user device, it is named Mac_Rx_Middle. Both these classes are added as pointers to the net devices in the *ns-3* AP and user device’s nodes. The net devices transmit method queue data received from the upper protocol stacks. The Mac_AP_Middle and Mac_Rx_Middle transmit method dequeue on the allocated slot and forward it down to the MAC low layer. For both LiFi AP and user devices, we provide a generic MAC layer called Mac_Low, responsible for managing access to the LiFi channel. It hosts a pointer to the middle layer mac and the physical layer objects of the AP. The Mac_Low class has a method for packet transmission and reception. In the transmission method, a header is put on the outgoing frame and sent down the channel. The header is removed from the frame in the receive method and checked for a control or a data frame and passed to the upper layer accordingly.
**Algorithm 1: TDMA resource sharing*****Initialize** slots array, slot time t, guard time G, and inter-frame spacing I* ***Label**: Start A Super Frame*   *Set number of control slots, T_c_*   *Calculate the total number of data slots, T, based on Equation (12)*   *Calculate frame_time T_f_ based on Equation (13) & super_frame_time T_s_ based on Equation (14)* ***Label**: Distribute Slots Based on QoS* ***for** i in Size (Users)*   *Allocate Slots based on Traffic Class (TC) according to Equation (11)*   ***if** Users [i] TC is AC_VO_*     *User.time_slots =*$n_{vo}*t$   ***else if** Users [i] TC is AC_VI_*     *User.time_slots =*$n_{vi}*t$   ***else***     *User.time_slots = t*   ***end***   ***end*** ***End*** ***Broadcast control Frame for Duration T_c,_ containing Sync Information*** **Label:***Start Transmitting Data Frame* **for** t **in**
*slots_array,*   *Select user, **u**, for transmission*   *Transmit user **u** Data*   *Wait for a Guard Time* ***end*** **End** ***Wait for a time equal to inter-frame gap G*** ***Label**: Start A Management Frame* *Send Beacon Packet in First Management Frame Slot with transmit power, MAC address, and IP address of the AP* *Wait for the Beacons Response in the Uplink Time Slot* *Add users with a response to Users array* *Remove from Users already associated users with no ACK*
***End*** **If**
*current_time >= T_s_*   **Goto**
*Start A Super Frame* **else**   **Goto**
*Transmit Data Frame* **end** **End**

### 4.4. Other Classes

We have provided helpers and other utility classes to facilitate the simulation of the LiFi networks. The helper classes facilitate building large-scale networks consisting of LiFi transmitters/APs configurations, including the user devices’ static association and integration with the WiFi. It also contains classes for easy configuration of the channels, physical layer, LiFi devices, and TDMA configurations. Apart from the helpers, we developed utility classes for MAC configuration and classes for performance analysis. 

## 5. Integration between LiFi and WiFi

The rapid fluctuations in the optical gain can result in frequent service outages. Therefore, the literature on LiFi strongly encourages its integration with other networking technologies such as WiFi, long-term evaluation (LTE), and/or 5G networks. This work considers the integration between LiFi and WiFi networks and leaves other networking technologies as future work. In Section 5.1, we provide a brief overview of the *ns-3* WiFi module, and in the later subsections, we discuss the different ways in which we can integrate LiFi and WiFi and address the associated challenges.

### 5.1. ns-3 WiFi

The WiFi has been standardized at the physical and MAC layer with regular standards updates to accommodate new features and technologies. The *ns-3* WiFi module provides an implementation of nearly all standards of IEE802.11, starting from 802.11a to 802.11ac. In *ns-3,* the physical layer models packet reception using two distinct techniques: Yet another simulator (Yans) model and a spectrum-based physical layer [35]. The MAC layer models the 802.11 distributed coordination function (DCF) for infrastructure (access point mode) and an Ad-hoc mode for direct communication between the user or infrastructure devices. The QoS support is provided through a distributed channel access (DCA) and an enhanced distributed coordination channel access (EDCA). In EDCA, four different queues are maintained for different QoS classes and each class has a mechanism for channel access based on QoS requirements. In our simulation, we have integrated the IEEE802.11ac version of WiFi. However, other standards could be trivially added to the framework. We use these existing capabilities of WiFi *ns-3* and our implementation of LiFi to provide a hybrid LiFi/WiFi system. The hybrid systems fall into two categories, namely asymmetric and symmetric. Both these arrangements are shown in Figure 1 where AP1 provides asymmetric communication while AP2 provides symmetric communication. Below we provide a detailed description of these integration mechanisms.

### 5.2. Asymmetric Integration between LiFi and WiFi

In the asymmetric arrangement, the LiFi downlink is provided through the VLC and the uplink via the WiFi. In addition to serving as an uplink to the LiFi users, the WiFi hosts’ other users and can be a potential landing network for the LiFi users when they experience connections drop. This asymmetric integration provides reliability because of the consistency of the WiFi signal. However, it can induce extra delay due to increased contention on the uplink, which can outweigh the LiFi benefits. Aside from this, several technical challenges need to be addressed before a fully functional system can be realized. Some of these challenges and their solutions are discussed below.

#### 5.2.1. Traffic Shaping at the Router

In traditional networks, the user devices use the same route for downlink and uplink communication. However, in the asymmetric hybrid system, the traffic destined to the LiFi network must be separated at the router and relayed to the LiFi AP before downlink transmission. For the results shown in Section 8, we have implemented a static routing table to separate traffic at the router, though these static routes can be replaced with dynamic routing. Each route in the static routing table consists of the destination IP, next hope interface IP, and the exit interface, and an example of this is shown in Table 1.

#### 5.2.2. Providing TCP and UDP Traffic over LiFi/WiFi Hybrid Systems

In the asymmetric hybrid systems, the WiFi is used as an uplink for the LiFi users including the establishment of TCP and UDP connections as well as for ACK transmissions/retransmissions. This arrangement poses a challenge for LiFi users, especially during the connection establishment. As we know, the TCP and UDP sockets listen to a remote server through the port and the IP interfaces used for connection establishment. Therefore, when the connection is requested through the WiFi uplink, and its response is relayed through the LiFi downlink to the user device, the listening socket will discard it because the response is received through a different IP interface and port address than the one used for connection establishment.

To address the above-mentioned challenge, we have used an address resolution protocol (ARP) spoofing mechanism; a similar approach has been adopted in the existing literature [36]. This method involves reconfiguring the user devices’ caches to have the LiFi’s AP as a default gateway and, therefore, store its IP address and MAC addresses as gateway information. Thus, when the user device initiates a TCP/UDP, it listens on the interface connected to the LiFi network. The outgoing packets from the LiFi user device are captured and forwarded via the WiFi net device. When the server responds, and the message is relayed by the router through the LiFi downlink and passed to the user device, it is successfully received on the sender socket. These steps are listed in the form of Algorithm 2 below.**Algorithm 2: Asymmetric TCP/UDP Traffic*****Initialize**: TCP/UDP Application connected to LiFi Interface* *Interface LiFi and WiFi Net Devices* ***while** 1 **do***    ***If** frame_size > MTU then*       ***continue***     ***end*** ***end*** *Change dest MAC (LiFi AP) addr to WiFi AP MAC addr* *Change source MAC addr to LiFi User Device MAC addr* *Compute IP checksum* *Compute UDP/TCP checksum* *Send Packet to WiFi AP* ***End***

### 5.3. Symmetric LiFi and WiFi Integration

In the symmetric hybrid system, we use LiFi to provide bidirectional communication. The downlink is provided through VLC while the uplink is supported using infrared technology. The symmetric systems can be trivially supported on the existing framework discussed in Section 4. The physical layer including the channel class remains the same; however, at the MAC layer, we allow for a resource scheduling scheme to reserve slots for the uplink communication and downlink communication. This arrangement allows users to be associated with one network or another, thus allowing both networks to be used to their full capability. It reduces the WiFi uplink’s contention, thus easing several challenges such as delayed feedback to the centralized controller that can result in outdated decisions in the network. 

## 6. Handover between LiFi and WiFi

The LiFi users are vulnerable to frequent service outages, especially when they are mobile; thus without a backup network, the standalone LiFi network can only support static users. To provide seamless connectivity to mobile users, an adequate handover mechanism is necessary that can land users on an appropriate AP/network. During the handover process, the current access point releases the share of resources allocated to the user, and the new network has to allocate resources to the users according to its service requirements. There are two broad categories of handovers in the LiF/WiFi hybrid systems: Horizontal handover (HHO) and vertical handover (VHO). In horizontal handover, the user is switched to AP of the same networking technology (e.g., LiFi to LiFi, WiFi to WiFi). On the other hand, in the vertical handover, users are associated with APs of different networking technology (e.g., LiFi to WiFi). In this work, we consider the vertical handover in which users are initially hosted by LiFi APs, and during connection, drop or are handed over to the WiFi. The horizontal handover can also be trivially supported on this framework and has been left for future work.

### 6.1. Handover Parameters

The decision to perform a handover is dictated by the performance metrics of the user device. Performance can be measured at the physical layer in terms of the packet error rate (PER), received signal strength (RSS), signal-to-noise ratio (SNR), and instantaneous throughput. The traditional approach is to monitor the threshold of the received signal strength, or any of the other metrics, from the current AP for a certain duration D. If during this duration the signal strength drops below a certain handover threshold, the handover process starts with disconnection from the serving AP, and connects to a new AP. This switching process takes a certain time that accounts for the network procedures to connect the user device to the target AP, and in the process, it can incur some packet loss.

### 6.2. Metric Monitoring Algorithm

A metric monitoring algorithm decides whether to perform a handover to a WiFi network or stay connected to the current network based on a periodic check of the user device’s performance metrics. Upon establishing a connection to the AP, the proposed algorithm starts recording metrics locally at a regular interval. For a user *i* associated with the LiFi *A_l_*, performance metrics are monitored over a duration *D*. During this *D*, the number of data packets received is represented as *P_s_*, where corrupted packets are represented by *P_c_*. The instantaneous throughput during *D* is represented by *T_i_*, which is the ratio of successfully delivered packets to the successfully received packets. Similarly, the instantaneous SNR is measured by the mean over duration *D*.

(15) $$T_{i}\;\;\;\;\;\;\;\;\;=\;\;\;\;\;\;\;\;\frac{P_{s}}{D}$$

(16) $$P_{err}\;\;\;\;\;\;=\;\;\;\;\;\;\;\;\;\;\frac{P_{s}}{P_{c}}$$

(17) $$SNR_{i\;\;\;\;\;}\;\;=\;\;\;\;\;\;\frac{\sum_{i=0}snr}{N}$$

where in the above equations $P_{c}$ is the number of packets corrupted, $P_{err}$ is the error rate, SNR_i_ is the mean signal strength during *D*, and *N* is the number of SNR measurements. 

At the physical layer of each device, the metric algorithm agent monitors all the critical metrics mentioned above. To decide whether to trigger handover, first the SNR and then the instantaneous throughput are checked for their predefined thresholds.

(18) $$SNR_{i}<\;\;\;SNR_{th}$$

(19) $$T_{i}<\;\;\;T_{th}$$

If the SNR and the instantaneous throughput during the specified duration is below a certain threshold, the user device should trigger a handover by requesting an association with another AP. If the signal strength is sufficient and the throughput is above a certain threshold, then the algorithm looks for packet loss ratio as it is possible that due to random orientation changes some packets can be dropped. A predetermined error threshold $P_{err\_th}\;$, based on the application requirement, is used to determine the activation of handover.

(20) $$P=\left\{\begin{array}{c} \;1;\;\;\;P_{error}>P_{err\_th}\\ o;\;\;\;\;\;\;\;\;\;\;\;\;\;\;\;\;otherwise \end{array}\right.$$

These handover operations are encapsulated into a metric monitoring Algorithm 3 given below. The metric monitoring algorithm has an agent in the user device that monitors the above-mentioned critical parameters. Once these parameters start to miss their threshold, the user device sends a request to the centralized controller to trigger a handover. The centralized controller in turn looks for the closest WiFi/LiFi APs and transfer the handover session to one of them.
**Algorithm 3: Handover*****Initialize**: Thresholds for performance metrics SNR_th_, T_th_, P_err_,* *instantaneous throughput, packet loss, mean_snr* ***Label**: Record Metrics at User Device* ***while** 1 **repeat***   *Based on Equation (15) calculate the instantaneous throughput*   *Base on Equation (16) calculates packet success probability*   *Based on Equation (17) calculate the mean SNR from the current AP* ***Goto** CheckMetrics* ***do** after time D* ***End*** ***Label**: Check Metrics* ***if** SNR < SNR_th_  or  P_err_  > P_th_  or*$T_{i}$*< T_th_*   ***return** 1* ***else***   ***return** 0* ***end*** **End**

## 7. Evaluation Metrics

The metrics-monitoring algorithm discussed above monitors instantaneous performance metrics at the physical layer for performing handover and ensuring continuous connectivity. However, to analyze the application-level performance gains from the hybrid LiFi/WiFi system, we evaluate it in terms of the packet delivery ratio (PDR), delay, throughput, and fairness between users.

### 7.1. Throughput

The throughput of WiFi and a LiFi station depends on packet size, SNR, and the physical layer rates. The per-station throughput in megabits per second (Mbps), *T*_LiFi/WiFi_, for WiFi/LiFi can be calculated as below:

(21) $$T_{\mathrm{LiFi}/\mathrm{WiFi}}=\frac{\left(P_{Rx}*8\right)}{e^{6}*\;T_{sim}}$$

where $T_{\mathrm{LiFi}/\mathrm{WiFi}}$ is the throughput and $P_{Rx}$ is the total received packets.

### 7.2. Delay

Delay is a sum of transmission queueing delay, propagation delay, and contention for time slots at WiFi/LiFi MAC layers, given in (18).

(22) $$Delay\;=\;\frac{Total\;Delay}{P_{Rx}}$$

### 7.3. Fairness between Users

We use Jain’s fairness index (JFI) to study the effects of increasing the number of users on individual users’ data rate performance [37].

(23) $$JFI\;=\;\frac{{\left(\sum x_{i}/O_{i}\right)}^{2}}{n\left(\sum x_{i}^{2}/O_{i}\right)}$$

where *O_i_* stands for optimal throughput of a single station.

### 7.4. Packet Delivery Ratio (PDR)

The packet delivery ratio is the number of packets transmitted and the number of packets received.

(24) $$\;PDR=\left(\frac{Tx_{packets}}{Rx_{packets}}\right)*100$$

## 8. Simulations

The LiFi framework has been evaluated for user’s mobility and the resulting handovers from it. Besides, the integrated LiFi/WiFi system is evaluated for a large number of users and its performance gains are measured in terms of throughput, delay, fair sharing, and PDR.

Three simulation scenarios are designed to demonstrate the potential usage of the LiFi *ns-3* framework. The first two scenarios are related to mobility and the handover capability, and the third one is related to the scalability aspect of the framework. In the first scenario, we consider a mobile user connected only to the LiFi network with no other network available for the handover. In the second scenario, we consider the static user and mobile users. The second-scenario users are first connected to the LiFi but can be dynamically associated with the WiFi based on the handover algorithm, presented in Algorithm 4. In the third simulation scenario, we demonstrate the improvements achieved for a LiFi/WiFi hybrid system in terms of system-wide performance metrics such as delay, fairness, and throughput. The parameters in Table 2 are used to setup LiFi and WiFi hybrid systems.

**Figure 3:**
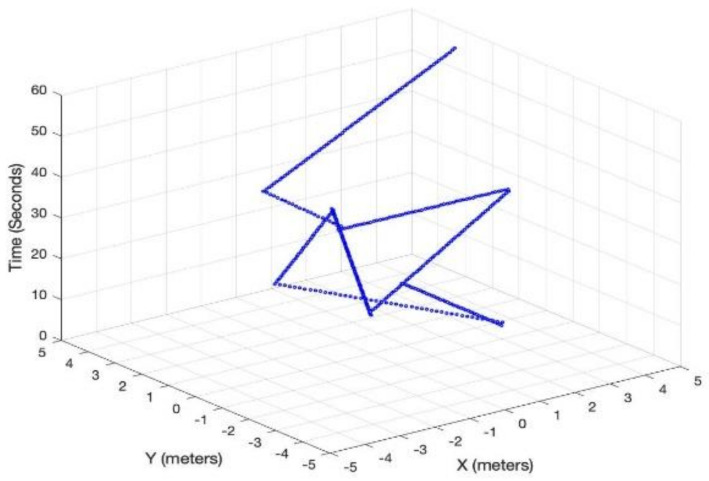
User mobility.

**Figure 4:**
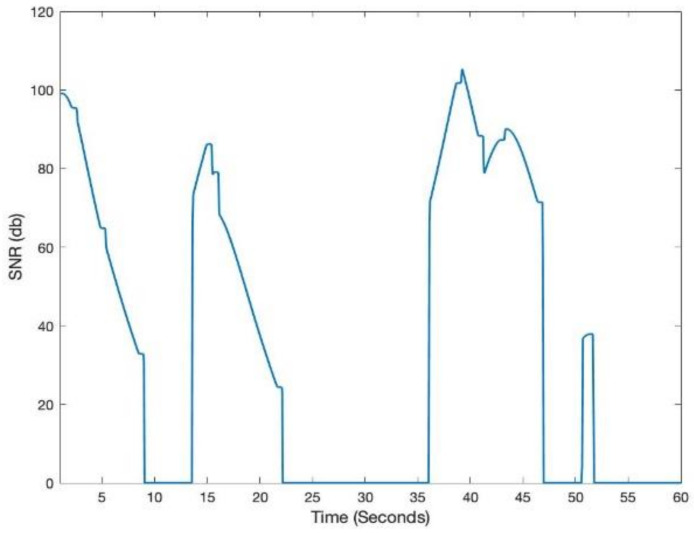
Instantaneous signal-to-noise ratio (SNR) for LiFi-only mobile user.

**Figure 5:**
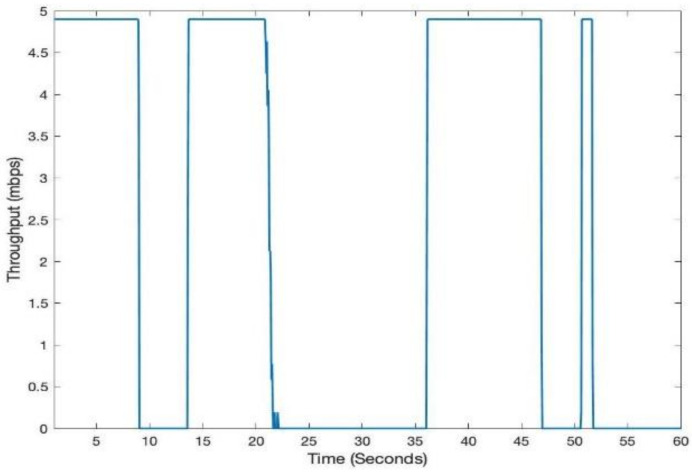
Instantaneous throughput for LiFi-only mobile user.

**Figure 6:**
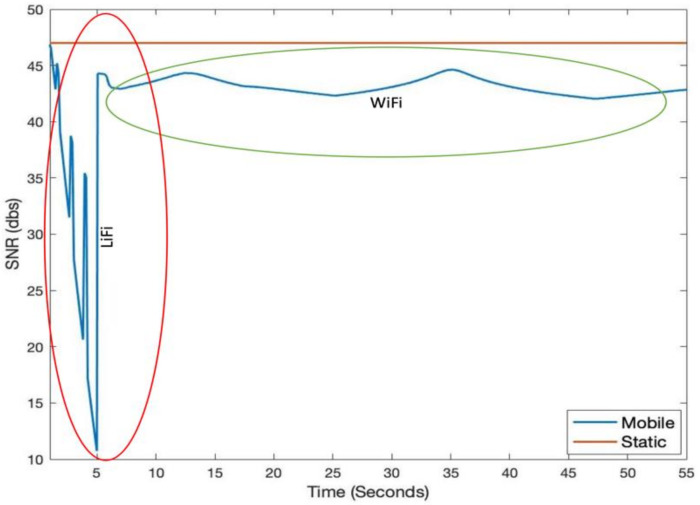
Instantaneous SNR for hybrid LiFi/WiFi system.

**Figure 7:**
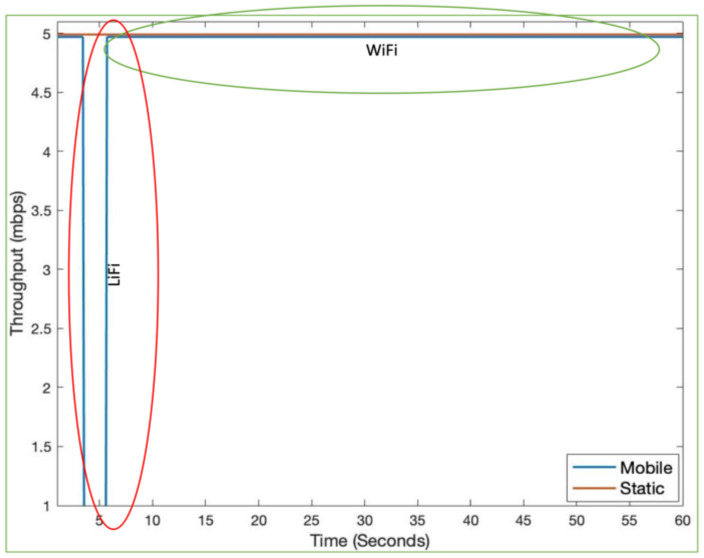
Instantaneous throughput for hybrid LiFi/WiFi.

Figure 3 shows LiFi mobile user’s movement in a room area of 10 m × 10 m. In addition to the user movement, we have modeled the user device’s orientation according to the discussion in Section 3.3. Figure 4 shows the SNR and throughput for the first scenario. The mobile user experiences drastic variations in short bursts in SNR due to the user’s random mobility. The user mobility accommodates for the twin effects i.e., the user device directionality vis-à-vis the LiFi AP, and the distance from it. The changes in SNR performance are reflected in the throughput of the user, as shown in Figure 5. Since the user is not provided with a backup network (e.g., WiFi), the handover is not triggered, and thus the performance metrics are significantly affected. For the second scenario, we have considered a mobile and static user. The mobile user is initially connected to the LiFi AP but once it starts moving around, the SNR and the corresponding data rates/throughput drop significantly as shown in Figure 6 and Figure 7, respectively. On the other hand, the static user has a consistent gain. At around 5 s into the simulation, the SNR and data rate are not enough to meet the user requirement, therefore the user device starts the handover process by sending a request to the centralized controller (CC). The CC completed the handover process by re-adjusting the routes in the LiFi and WiFi APs and takes some time before the user SNR and data rates improve. Since the user is not connected to either of the networks during the handover process, it can incur some packet loss. However, although there is some packet loss, the instantaneous parameters-based handover ensures that the user device has connectivity, even if it is mobile and experiencing sudden fluctuations in SNR. This shows that when we provide LiFi with a backup system in the form of WiFi, its performance metrics improve significantly. 

**Figure 8:**
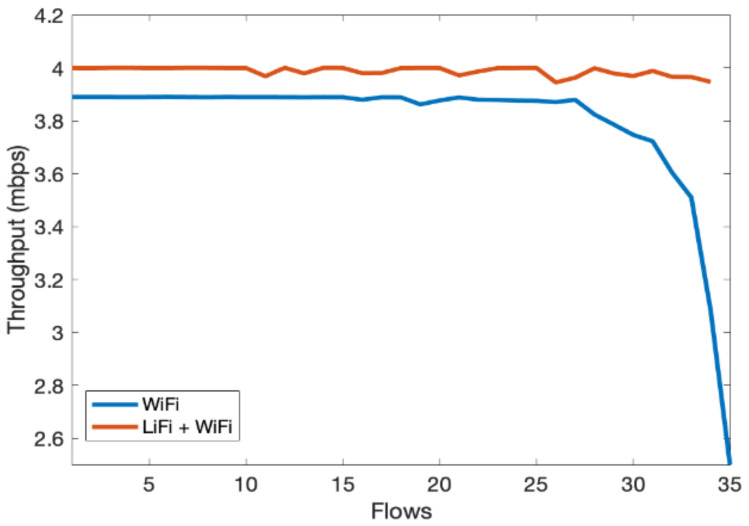
Per-user throughput vs. number of flows.

**Figure 9:**
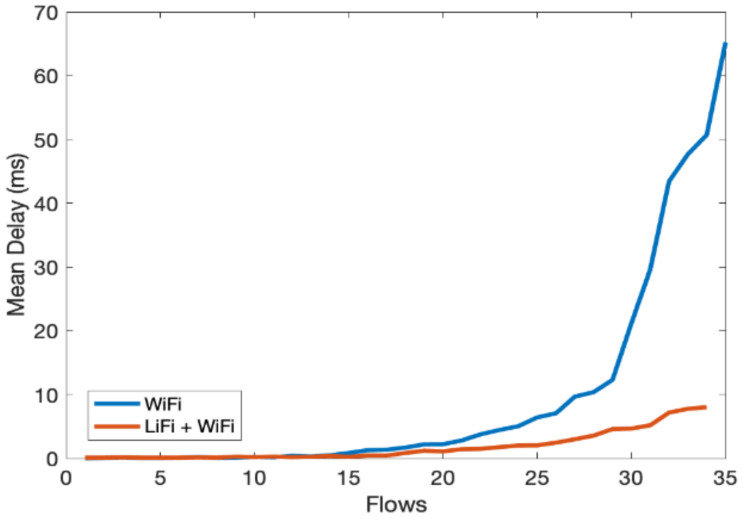
Average delay vs. number of flows.

**Figure 10:**
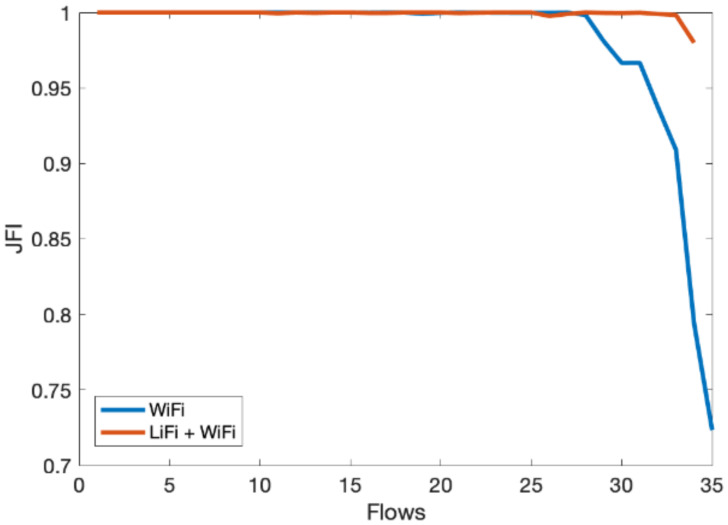
Jain’s fairness index (JFI) vs. number of flows.

**Figure 11:**
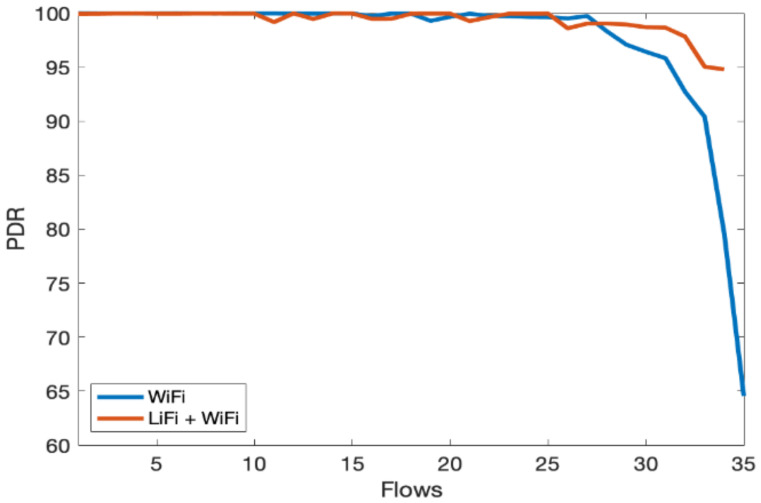
Packet drop ratio (PDR) vs. number of flows.

In the third scenario, we evaluate the overall performance gain of the hybrid LiFi and WiFi network. We consider an indoor network consisting of multiple users with Flows ranging from 1 to 35. Flows are unique TCP/UDP connections and are widely used in the literature to represent users; therefore, users and flows are exchangeable. We have limited one flow to one physical user. However, multiple flows can originat from a single user and it will have the same effect. The throughput of the hybrid system is shown in Figure 8, which shows significant performance gain over the WiFi-only system as the contention over WiFi channels decreased due to traffic offloads to LiFi. In Figure 9, similar performance gains can be observed for per-user delay vis-à-vis increase in the number of flows. Figure 10 shows that the fairness index remains stable with an increase in the number of flows in the hybrid network. Lastly, PDR results show that the hybrid system can avoid congestion due to multiple contending stations for the network resources, as shown in Figure 11. As the number of users increases, the WiFi-only system starts dropping packets, while in comparison, the hybrid system delivers much more of the packets successfully to the network end users. The difference in PDR becomes more pronounced as the number of users increases beyond 15.

We have only evaluated certain aspects of the framework, but researchers can add several research applications such as handover optimization, comparison between symmetric and asymmetric systems, advanced optimization algorithms for the MAC layer, and admission control schemes. The source code of the simulation framework can be found in [14].

## 9. Conclusions

We have developed an open-source LiFi framework in *ns-3* to simulate an indoor LiFi/WiFi network in this work. Our simulation modules provide an implementation of the physical and MAC layers. It also allows for multiuser access with QoS-based resource sharing and provides mobility support and dynamic user association. More importantly, it monitors critical performance metrics to trigger handover between LiFi and WiFi. We have simulated the handover capability, the homogeneous WiFi, and the hybrid LiFi/WiFi system for critical network parameters such as throughput, delay, fairness, and PDR. The source code is made available to the research community, which can be modified and extended as per their requirement. The availability of source code would facilitate new researchers to analyze the hybrid LiFI system holistically.

## Figures and Tables

**Table 1:** Traffic shaping.

Destination IP	Next Hope IP	Exit Interface
192.168.0.2	192.168.1.1	1

**Table 2:** WiFi Simulation parameters.

Parameters	Value
Tx Power	20 dBm
Bandwidth Per WiFi Channel	80 MHz
PSD of Noise	−174 dBm/Hz
DIFS	32 μs
WiFi Slot time	9 μs
SIFS	16 μs
LiFi slot time	16 μs
Guard band	20 μs
Interframe spacing	10 μs
WiFi Modulation and Coding Scheme (MCS)	7
LiFi Modulation scheme	OOK
WiFi Rate Control Algorithm	VHT
